# Supplementary material for: Clinical characteristics and outcomes of 952 hospitalized COVID-19 patients in The Netherlands: A retrospective cohort study
Source: PLoS One. 2021 Mar 18;16(3):e0248713. doi: 10.1371/journal.pone.0248713 (PMC7971488; doi:10.1371/journal.pone.0248713)
Supplement: S1 Data — (DOCX) [file pone.0248713.s001.docx]

S1 Tables. Characteristics, comorbidities and outcomes in patients stratified by age, split in subgroups by ICU admission policies or ICU admission.

S1 Figure. Flow chart of included patients. (supplementary).
S2 Figure. Survival curve of COVID-19 patients stratified by age.
S3 Figure. Survival curve of COVID-19 patients aged >70 years stratified by ICU policy.

This supplementary material has been provided by the authors to give readers additional information about their work.

**S1 Table a.** Characteristics, comorbidities and outcomes in patients stratified by age, split in subgroups by ICU admission policies or ICU admission.

|  | Patients aged 70 years or older (n=473) | | | | | |
| --- | --- | --- | --- | --- | --- | --- |
|  | Admitted to ward with no ICU restrictions (n=140) | | Admitted to ward with non ICU admission policy (n=269) | | Admitted to ICU (n=64) | |
| **Characteristic** - n (%) | Survived 112 (80.0%) | Deceased 28 (20.0%) | Survived 134 (49.8%) | Deceased 135 (50.2%) | Survived 33 (51.6%) | Deceased 31 (48.4%) |
| Median age - (IQR) | 74 (72-78) | 78 (75-81) | 80 (75-83) | 80 (76-84) | 72 (71-75) | 74 (73-78) |
| Gender, male | 77 (68.8) | 21 (75.0) | 82 (61.2) | 86 (63.7) | 22 (66.7) | 27 (87.1) |
| Cardiovascular diseases | 76 (67.9) | 19 (67.9) | 107 (79.9) | 109 (80.7) | 23 (69.7) | 22 (71.0) |
| Pulmonary diseases | 22 (19.6) | 9 (32.1) | 45 (33.6) | 42 (31.1) | 7 (21.2) | 6 (19.4) |
| Solid organ malignancy | 19 (17.0) | 5 (17.9) | 25 (18.7) | 27 (20.0) | 6 (18.2) | 6 (19.4) |
| Immunocompromised | 23 (20.5) | 10 (35.7) | 29 (21.6) | 29 (21.5) | 4 (12.1) | 9 (29.0) |
| Cerebrovascular disease | 13 (11.6) | 5 (17.9) | 36 (26.9) | 29 (21.5) | 4 (12.1) | 3 (9.7) |
| Diabetes | 32 (28.6) | 6 (21.4) | 35 (26.1) | 40 (29.6) | 10 (30.3) | 9 (29.0) |
| Chronic kidney disease without RRT | 10 (8.9) | 5 (17.9) | 33 (24.6) | 22 (16.3) | 1 (3.0) | 4 (12.9) |
| Median length of hospital stay (IQR) | 5 (3-9) | 5 (4-7) | 7 (4-10) | 5 (3-7) | 31 (26-49) | 17 (8-24) |
| Median length of ICU stay (IQR) | 0 (0) | 0 (0) | 0 (0) | 0 (0) | 22 (9-34) | 12 (6-22) |
| Abbreviations: ICU, intensive care unit; IQR, interquartile range; BMI, body mass index; RRT, renal replacement therapy. | | | | | | |

**S1 Table b.**

|  | Patients aged 61 to 69 years or older (n=205) | | | | | |
| --- | --- | --- | --- | --- | --- | --- |
|  | Admitted to ward with no ICU restrictions (n=117) | | Admitted to ward with non ICU policy (n=29) | | Admitted to ICU (n=59) | |
| **Characteristic** - n (%) | Survived 116 (99.1%) | Deceased 1 (0.9%) | Survived 16 (55.2%) | Deceased 13 (44.8%) | Survived 44 (74.6%) | Deceased 15 (25.4%) |
| Median age - (IQR) | 66 (63-68) | 65 (65-65) | 67 (64-68) | 67 (65-69) | 67 (64-68) | 67 (65-67) |
| Gender, male | 64 (55.2) | 1 (100) | 9 (56.3) | 8 (61.5) | 32 (72.7) | 10 (66.7) |
| Cardiovascular diseases | 64 (55.2) | 1 (100) | 11 (68.8) | 11 (84.6) | 29 (65.9) | 9 (60.0) |
| Pulmonary diseases | 23 (19.8) | 1 (100) | 8 (50.0) | 6 (46.2) | 8 (18.2) | 0 (0) |
| Solid organ malignancy | 14 (12.1) | 0 (0) | 5 (31.3) | 3 (23.1) | 7 (15.9) | 2 (13.3) |
| Immunocompromised | 25 (21.6) | 0 (0) | 3 (18.8) | 6 (46.2) | 8 (18.2) | 4 (26.7) |
| Cerebrovascular disease | 9 (7.8) | 0 (0) | 1 (6.3) | 4 (30.8) | 3 (6.8) | 0 (0) |
| Diabetes | 27 (23.3) | 1 (100) | 4 (25.0) | 3 (23.1) | 4 (9.1) | 3 (20.0) |
| Chronic kidney disease without RRT | 6 (5.2) | 1 (100) | 4 (25.0) | 1 (7.7) | 1 (2.3) | 1 (6.7) |
| Median length of hospital stay (IQR) | 5 (3-9) | 8 (8-8) | 9 (4-14) | 5 (3-7) | 23 (35-52) | 15 (4-28) |
| Median length of ICU stay (IQR) | 0 (0) | 0 (0) | 0 (0) | 0 (0) | 21 (13-32) | 13 (4-26) |
| Abbreviations: ICU, intensive care unit; IQR, interquartile range; BMI, body mass index; RRT, renal replacement therapy. | | | | | | |

**S1 Table c.**

|  | Patients aged 60 years or under (n=274) | | | | | |
| --- | --- | --- | --- | --- | --- | --- |
|  | Admitted to ward with no ICU restrictions (n=203) | | Admitted to ward with non ICU policy (n=11) | | Admitted to ICU (n=60) | |
| **Characteristic** - n (%) | Survived 202 (99.5%) | Deceased 1 (0.5%) | Survived 6 (54.5%) | Deceased 5 (45.6%) | Survived 50 (83.3%) | Deceased 10 (16.7%) |
| Median age - (IQR) | 52 (48-56) | 49 (49-49) | 56 (49-58) | 57 (55-59) | 53 (47-58) | 56 (48-59) |
| Gender, male | 115 (56.9) | 0 (0) | 4 (66.7) | 4 (80.0) | 35 (70.0) | 8 (80.0) |
| Cardiovascular diseases | 52 (25.7) | 1 (100) | 4 (66.7) | 3 (60.0) | 18 (36.0) | 0 (0) |
| Pulmonary diseases | 47 (23.3) | 1 (100) | 1 (16.7) | 2 (40.0) | 7 (14.0) | 3 (30.0) |
| Solid organ malignancy | 13 (6.4) | 0 (0) | 1 (16.7) | 1 (20.0) | 5 (10.0) | 1 (10.0) |
| Immunocompromised | 39 (19.3) | 1 (100) | 0 (0) | 1 (20.0) | 9 (18.0) | 4 (40.0) |
| Cerebrovascular disease | 6 (3.0) | 0 (0) | 1 (16.7) | 1 (20.0) | 2 (4.0) | 0 (0) |
| Diabetes | 27 (13.4) | 0 (0) | 2 (33.3) | 1 (20.0) | 10 (20.0) | 1 (10.0) |
| Chronic kidney disease without RRT | 6 (3.0) | 0 (0) | 0 (0) | 0 (0) | 1 (2.0) | 0 (0) |
| Median length of hospital stay (IQR) | 4 (3-7) | 3 (3-3) | 6 (5-10) | 9 (2-12) | 25 (17-39) | 25 (17-30) |
| Median length of ICU stay (IQR) | 0 (0) | 0 (0) | 0 (0) | 0 (0) | 14 (9-21) | 23 (12-26) |
| Abbreviations: ICU, intensive care unit; IQR, interquartile range; BMI, body mass index; RRT, renal replacement therapy. | | | | | | |

**S1 Figure.** Flow chart of included patients.


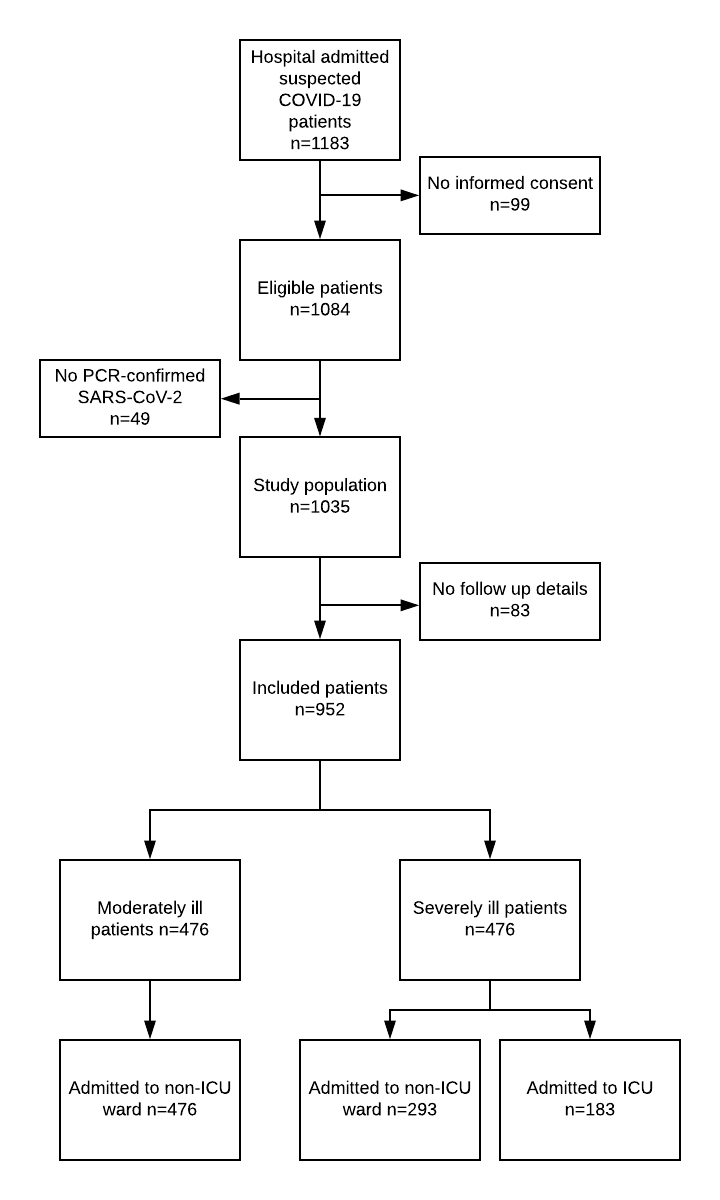


S1 Figure. Flow chart of included patients. Patients with no confirmed PCR or no follow-up details were excluded. Included patients were divided according to disease severity: moderately ill and severely ill. The severely ill patient group wqs subdivided into those admitted to ICU and non-ICU wards.

**S2 Figure.** Survival curve of COVID-19 patients stratified by age.


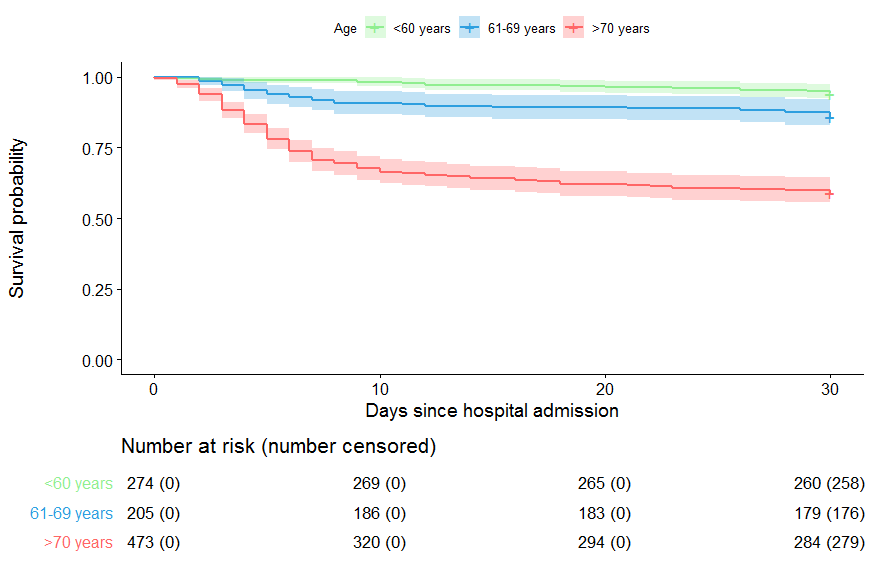


S2 Figure. Kaplan-Meier survival curve of in-hospital COVID-19 patients stratified by age. No adjustments for confounders were made. Patients that were discharged with palliative care (n=5), were considered to be deceased at day 30. An assumption is made that if patients were discharged with the reason of clinical improvement before day 30, patients were still alive at day 30 (right point imputation). The probability of COVID-19 related death after discharge due to clinical improvement is very low, especially since hospital re-admissions are registered and incorporated in these data. Nevertheless, there is a small risk of bias considering the right point imputation. Therefore, this Kaplan-Meier curve should be used descriptively and exploratively.

**S3 Figure.** Survival curve of COVID-19 patients aged >70 years stratified by ICU policy.


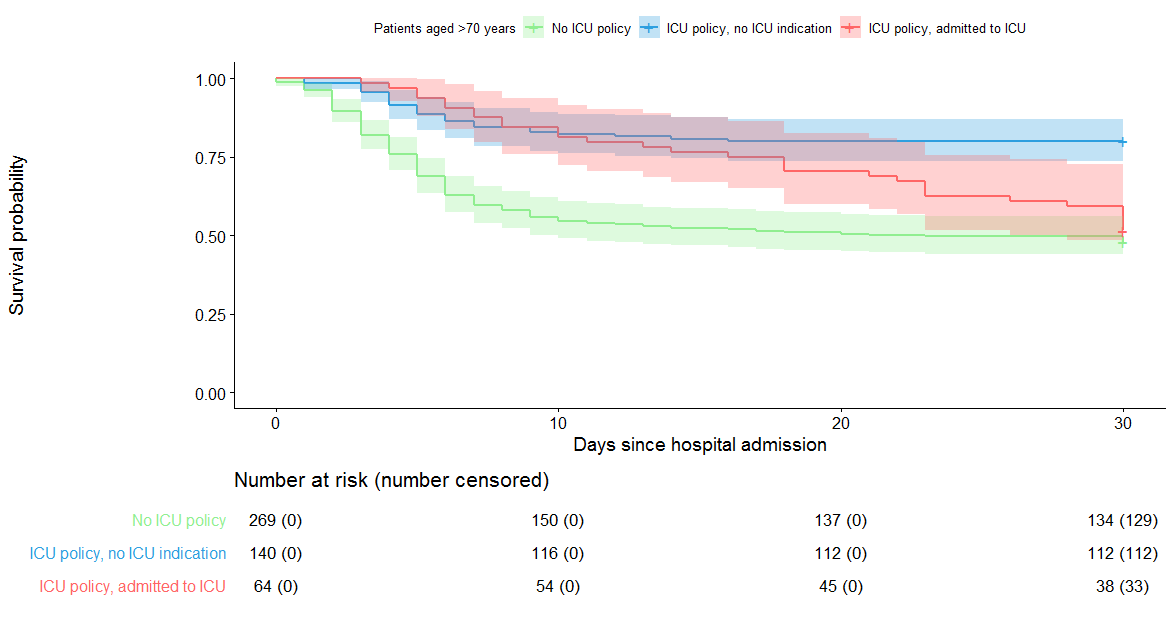


S3 Figure. Kaplan-Meier survival curve of in-hospital COVID-19 patients stratified by ICU admission policy. No adjustments for confounders were made. Patients that were discharged with palliative care (n=5), were considered to be deceased at day 30. An assumption is made that if patients were discharged with the reason of clinical improvement before day 30, patients were still alive at day 30 (right point imputation). The probability of COVID-19 related death after discharge due to clinical improvement is very low, especially since hospital re-admissions are registered and incorporated in these data. Nevertheless, there is a small risk of bias considering the right point imputation. Therefore, this Kaplan-Meier curve should be used descriptively and exploratively.
