# Supplementary material for: Clinical characteristics and outcomes of 952 hospitalized COVID-19 patients in The Netherlands: A retrospective cohort study
Source: PLoS One. 2021 Mar 18;16(3):e0248713. doi: 10.1371/journal.pone.0248713 (PMC7971488; doi:10.1371/journal.pone.0248713)
Supplement: Attachment — Submitted filename: Response to Reviewers.docx [file pone.0248713.s002.docx]

To: Editorial Board

*PLOS One*

Nijmegen, 6 February, 2021

We hereby resubmit our manuscript entitled ‘Clinical characteristics and outcomes of 952 COVID-19 patients: a retrospective cohort study’. We have appreciated the comments made by the reviewers and have made changes in the manuscript according to their suggestions. Furthermore, we uploaded an anonymized minimal dataset to replicate our study findings as a supporting data file.

Lastly, as suggested by reviewer two, we now describe more explicitly our outcome data of patients over 70 years of age with or without an ICU admission policy and added a Figure S3 to the Supplementary. Our preference would be to take up Figure S2 and S3 in the main article, however we could not pursue because of the Table and Figure limitations as determined by *Plos One*. We leave it up to the editor of *Plos One* to decide on this matter.

We hope our manuscript is now suitable for publication in *PLOS One*.

Yours sincerely,

Niels Pouw and Jacobien Hoogerwerf

**Editors’ Comments:**

**Journal requirements:**

When submitting your revision, we need you to address these additional requirements.

**Comment 1.** Please ensure that your manuscript meets PLOS ONE's style requirements, including those for file naming. The PLOS ONE style templates can be found at
<https://journals.plos.org/plosone/s/file?id=wjVg/PLOSOne_formatting_sample_main_body.pdf> and
<https://journals.plos.org/plosone/s/file?id=ba62/PLOSOne_formatting_sample_title_authors_affiliations.pdf>

**Response 1.** The main body and author affiliations now meet PLOS ONE’s style requirements, including those for file naming.

**Comment 2.** In the Methods, please clarify that participants provided oral consent. Please also state in the Methods:
- Why written consent could not be obtained
- Whether the Institutional Review Board (IRB) approved use of oral consent
- How oral consent was documented

For more information, please see our guidelines for human subjects research: <https://journals.plos.org/plosone/s/submission-guidelines#loc-human-subjects-research>

**Response 2.** According to our institutional review board (RIB), written consent was not necessary and therefore approved the use of oral consent for this study. Oral consent was obtained from all patients or their family and documented in the electronical medical records. We addressed this now more clearly in the ethical considerations section in the Method.

**Comment 3.** In the ethics statement in the manuscript and in the online submission form, please provide additional information about the patient records/samples used in your retrospective study, including:

a) whether all data were fully anonymized before you accessed them;

b) the date range (month and year) during which patients' medical records/samples were accessed.

**Response 3.**
a) All data were entered anonymously into the electronic case report forms (eCRF; Castor Electronic Data Capture). The authors that were involved in the data analysis only received coded data without traceable information leading to patients involved in this study.

b) The first time that medical records of patients included in this study were accessed, was March 17th, 2020. The last time was the same date as when the follow up was halted (1^st^ of July).

We clarified both a) and b) in the manuscript (data collection).

4. We note that you have indicated that data from this study are available upon request. PLOS only allows data to be available upon request if there are legal or ethical restrictions on sharing data publicly. For information on unacceptable data access restrictions, please see <http://journals.plos.org/plosone/s/data-availability#loc-unacceptable-data-access-restrictions>.

In your revised cover letter, please address the following prompts:

a) If there are ethical or legal restrictions on sharing a de-identified data set, please explain them in detail (e.g., data contain potentially identifying or sensitive patient information) and who has imposed them (e.g., an ethics committee). Please also provide contact information for a data access committee, ethics committee, or other institutional body to which data requests may be sent.

b) If there are no restrictions, please upload the minimal anonymized data set necessary to replicate your study findings as either Supporting Information files or to a stable, public repository and provide us with the relevant URLs, DOIs, or accession numbers. Please see <http://www.bmj.com/content/340/bmj.c181.long> for guidelines on how to de-identify and prepare clinical data for publication. For a list of acceptable repositories, please see <http://journals.plos.org/plosone/s/data-availability#loc-recommended-repositories>.

We will update your Data Availability statement on your behalf to reflect the information you provide.

**Response 4.** We uploaded a Supporting Information file with the minimal required anonymized data to reproduce our study findings.

5. PLOS requires an ORCID iD for the corresponding author in Editorial Manager on papers submitted after December 6th, 2016. Please ensure that you have an ORCID iD and that it is validated in Editorial Manager. To do this, go to ‘Update my Information’ (in the upper left-hand corner of the main menu), and click on the Fetch/Validate link next to the ORCID field. This will take you to the ORCID site and allow you to create a new iD or authenticate a pre-existing iD in Editorial Manager. Please see the following video for instructions on linking an ORCID iD to your Editorial Manager account: <https://www.youtube.com/watch?v=_xcclfuvtxQ>

**Response 5.** The corresponding author, Jacobien Hoogerwerf, validated her ORCID iD to this submission in Editorial Manager.

6. Please include captions for your Supporting Information files at the end of your manuscript, and update any in-text citations to match accordingly. Please see our Supporting Information guidelines for more information: [http://journals.plos.org/plosone/s/supporting-information](about:blank)

**Response 6.** We included captions for our supporting files at the end of the manuscript, according to the PLOS One supporting information guidelines.

Additional Editor Comments:

dear Authors follow reviewer suggestion to improve your paper

**Reviewer comments:**

**Reviewer #1:** 
I read with great interest the manuscript. Authors wrote a very good article.
Only some minor suggestion

**Minor Comments:**

**Comment 1.** Introduction: update data on global burden of SARS CoV2 infection. Methods and result are clear. Well tables and statistical analysis

**Response 1.** We thank the referee for carefully examining our manuscript. We updated the data of the global burden of SARS-CoV-2 at the last possible time before submission (6 February, 2021) and changed this in the manuscript accordingly.

**Comment 2.** Discussion: discuss better the pharmacological approach also in other European countries ( see and citeUse of hydroxychloroquine in hospitalised COVID-19 patients is associated with reduced mortality: Findings from the observational multicentre Italian CORIST study. Eur J Intern Med. 2020 Dec;82:38-47. doi: 10.1016/j.ejim.2020.08.019. Epub 2020 Aug 25. PMID: 32859477; PMCID: PMC7446618 and RAAS inhibitors are not associated with mortality in COVID-19 patients: Findings from an observational multicenter study in Italy and a meta-analysis of 19 studies. Vascul Pharmacol. 2020 Dec;135:106805. doi: 10.1016/j.vph.2020.106805. Epub 2020 Sep 28. PMID: 32992048; PMCID: PMC7521934.. Compare your data with comorbidites in other big data article Common cardiovascular risk factors and in-hospital mortality in 3,894 patients with COVID-19: survival analysis and machine learning-based findings from the multicentre Italian CORIST Study. Nutr Metab Cardiovasc Dis. 2020 Oct 30;30(11):1899-1913. doi: 10.1016/j.numecd.2020.07.031. Epub 2020 Jul 31. PMID: 32912793 )

**Response 2.** This is a description of a large population in the first months of the COVID-19 pandemic, whereas most people (around 70%) in this cohort were treated with chloroquine and only a small portion with hydroxychloroquine. Based on several important early published observational studies, chloroquine and hydroxychloroquine later became banned as a treatment option (Geleris et al. NEJM 2020, DOI: 10.1056/NEJMoa2012410; Mahevas et al. MBJ 2020, DOI:10.1136/bmj.m1844 and Hernandez et al. Ann Intern Med 2020, DOI: 10.7326/M20-2496). However, discussing the possible benefits of hydroxychloroquine in COVID-19 patients is still subject to debate at this time and randomized controlled trials are warranted before we can draw a final conclusion about the effect of hydroxychloroquine in treating COVID-19 patients.

In respect to the comments of both reviewers (reviewer #1 comment 2 and reviewer #2 comment 6), we do realize that received treatment is still important information for an extensive description of our cohort and desirable when comparing to other studies. As a result, we added these data to table 2 and 3 and described the main outcomes in the results section of the manuscript as follows:

“In this cohort, 664 (69.7%) patients were treated predominantly with chloroquine. Hydroxychloroquine, Remdesivir, lopinavir/ritanovir and Anakinra were used scarcely at the time. A quarter of all patients received no medication.”

and

“At the ICU, most patients received invasive ventilation 129 (70.5%) and otherwise non-invasive ventilation 57 (31.1%).”

Furthermore, as suggested by the reviewer, we compared our comorbidities with the CORIST study and concluded that the high prevalence of comorbidities in hospitalized COVID-19 patients was the same in both studies. Therefore, we mentioned this in the discussion of our manuscript:

“COVID-19 in hospitalized patients in The Netherlands, proved to be a disease of older age and the prevalence of comorbidities were high in this population, congruent with the findings of a large observational study in Europe.[21]”

**Comment 3.** Furthermore add data on policy and lockdown during pandemic

**Response 3.** We agree with the referee and added information about the lockdown during the pandemic in the Introduction section as follows: **“**During the first COVID-19 wave in the Netherlands, in times of movement restrictions and a (partial) lockdown, physicians were encouraged to discuss the ICU admission policy with their patients.”

**Reviewer #2:** 
Pouw and colleagues are to be commended for their accurate ISARIC-compliant descriptive work on 952 patients with coronavirus 2 disease. Although the findings are clearly reported and an interesting longitudinal follow up is performed on several variables, here are some few points that need to be addressed:

**Major Comments:**

**Comment 1.** It is not clear if the study was registered

**Response 1.** Firstly, we would like to thank the reviewer for carefully examining our manuscript. Our answers and ensuing changes in the manuscript are as follows:
This study was not registered, because of the retrospective nature of this study. However, in hindsight there is no reason not to register observational retrospective studies and could only have benefits for the scientific community and its stakeholders (Williams et al. CMAJ; doi: [10.1503/cmaj.092252](https://dx.doi.org/10.1503%2Fcmaj.092252)).

**Comment 2.** Two groups are presented in the statistical anlysis (severe vs. moderate), but the reason for analysing based on these two groups should be stated earlier, and also moderate disease should be defined.

**Response 2.** We totally agree with the referee that moderately ill patients should be defined. We added the following to the definitions section of the Methods: “Moderately ill patients were all other patients that were hospitalized in this cohort, but did not meet the requirements of severely ill patients stated above.” Furthermore, we stated the reason for analyzing the two groups earlier, at the end of the Introduction section.

**Comment 3.** Of the patients admitted to ICU it is not clear how many received invasive or non-invasive ventilation

**Response 3.** We agree with the referee that data in regard to ventilation state is valuable information, especially for patients admitted to ICU. Therefore we added ventilation data to Table 3 (page 11) and added the following in the results section (page 12):”At the ICU, most patients received invasive ventilation 129 (70.5%) and otherwise non-invasive ventilation 57 (31.1%).”

**Comment 4.** The one third of patients with no-ICU admission policy is presented as a key finding, and it is actually the finding that makes this paper more interesting compared to other retrospective cohorts. Althouth TAble 4 and Supp. table 1 try to cover this I believe the characteristics of the patients that chose to go for a non-icu admission could be better exposed. E.g. did they present with different vital signs? The interesting finding that above 70yrs mortality is the same is also important but seems undersnowed now. Also, no mention to the non-ICU admission policy is made in the paper conclusions. E.g. A figure like S2 but considering groups based on ICU admission policy would be interesting. I am not suggesting to conduct data-driven reporting, just that a salient finding compared to many others cohort studies be better explored and conveyed.

**Response 4.** We completely agree with the referee that our findings regarding patients with a non-ICU admission policy are interesting and could be better explored and conveyed.

We completely agree with the referee that the above 70 years mortality in both ICU and in non-ICU admission policy groups is an important finding, but could be emphasized more appropriately in this manuscript. Therefore we added an additional figure (Figure S3), depicting mortality in patients aged 70 years and over with an ICU- or non-ICU admission policy, as suggested by the reviewer. Figure S3 shows that patients aged 70 years and over with a non ICU admission policy compared to patients aged 70 years and over admitted to ICU had similar survival after 30 days. Figure S3 also demonstrates that death occurs early in the course of hospital admission in patients above 70 years with a non ICU admission. Possibly, a non ICU admission policy prevented the patient burden they might have experienced if they were admitted to an ICU. We stated this in the Results section as follows:

“Furthermore, we visualized the survival of patients aged 70 years and over based on ICU admission policy (Fig S3). Patients aged 70 years and over with a non ICU admission policy show similar survival after 30 days compared to patients of this age group admitted to the ICU. Moreover, patients above 70 years deceased early during the course of hospital admission as compared to patients in this age group who were admitted to the ICU.”

We added the following to the Discussion section:

“In this manuscript, we demonstrate similar survival rates between patients above 70 years admitted to the ICU compared to patients in the same age group with a non ICU admission policy. In the latter group many patients deceased early during the course of their hospital admission, thereby possibly preventing patient burden that would have occurred if they were admitted to an ICU. Knowledge of patient characteristics, clinical outcomes, prognostic factors and course of disease are required to more distinctively identify factors that could help to differentiate which COVID-19 patients benefit from ICU admission.”

We added the following to the Conclusion section:

“Hospitalized COVID-19 patients aged >70 years had high mortality and a large group of patients aged over 70 years were admitted to the ward because of a non ICU admission policy. This group had similar mortality rates compared to patients over 70 years who were admitted to the ICU. Mortality occurred earlier in the course of hospital admission in the first group, reflecting the possible benefit of being spared a high burden of disease which comes with an ICU admission.”

**Comment 5.** Two groups are shown in figure 1, but it is not clear if a longitudinal comparison wants to be tested (then should be added to methods). e.g. is CRP consistently different among the groups or not? when saying that Ddimer changes after 15 days, no hypothesis was tested there, should we only eyeball?

**Response 5.** The objective of Figure 1 was to give global insights of laboratory parameters between moderately and severely ill COVID-19 patients during hospitalization. Specific longitudinal tests were not performed. Instead, we showed our data with 95% confident intervals for each consecutive day based on symptom duration. When 95% confident intervals don’t overlap, a significant difference is observed. Statements in this manuscript were based on these confident intervals on certain time points during hospitalization.

**Comment 6.** Although for some reason received treatment is not an endpoint of the study, it would help to know major things like use of oxygen and NIV in the two groups.

**Response 6.** We would like to refer to the reply from reviewer #1, comment 2. Following both recommendations we added our treatment data in table 2 and 3 and the corresponding text in the results.

**Comment 7.** In discussion the findings related to ICU admissions could be compared to the findings of the PRoVENT-COVID study performed in the same country, to see if the findings from the hospitals in the two regions participating in the study are comparable to the wider country.

**Response 7.** We completely agree with the referee that we should discuss this study in our manuscript, to see if the findings are comparable to the wider country. Both cohorts were similar in median age, gender distribution and roughly the same mortality rates were found. We state this in the discussion session as followed:

“In the PRoVENT-COVID study, also conducted in The Netherlands and with an ICU population with matching patient characteristics (age and gender distribution), ICU mortality rates were roughly equal. [30]”

**Comment 8.** Very few study limitations are presented.

**Response 8.** We agree with the referee that more study limitations should be presented to allow a meaningful interpretation with all strengths and weaknesses of the work. We have included new text in the discussion to point out some limitations. We hope we have thereby addressed this concern of the reviewer.

**Comment 9.** For longitudinal data very little info is provided on how it was collected ("every 48-72h" is rather imprecise and hampers replicability). And for vital signs collected more than once a day, were the worse used, or the best, etc.? Please add some details for clarity. Also, vital signs collected are not reported anywere it seems.

**Response 9.** We agree with the referee that the method of data collection regarding our longitudinal data could be more accurately described. The blood samples were collected 3-4 times per week (thus around every 48-72 hours). For laboratory values and vital signs on admission, the first documented value was entered in Castor. We now clarified this in the study procedures in the Methods section.

We collected vital signs on admission but we also had data of daily vital signs during hospitalization. We considered showing the vital signs in longitudinal data graphs, but we did not because our clinical observation was that vital signs frequently deteriorated suddenly instead of gradually. So, we felt that depicting daily vital signs longitudinally would not reflect the real situation.

**Minor Comments:**

**Comment 1.** line 182: please reword (repetitive use of "most common", "more frequently")

**Response 1.** In accordance with the suggestion of the reviewer, we rephrased some sentences, increasing the readability of the manuscript.
